# Short sleeping duration is associated with a higher risk of asymmetric handgrip strength among older Chinese males: a cross-sectional study evidence from the China health and retirement longitudinal study

**DOI:** 10.3389/fpubh.2023.1246008

**Published:** 2023-10-05

**Authors:** Yilin Wang, Mei Li, Xiaoyan Chen

**Affiliations:** ^1^Zigong Affiliated Hospital of Southwest Medical University, Zigong Psychiatric Research Center, Zigong, Sichuan Province, China; ^2^Zigong Institute of Brain Science, Zigong, Sichuan Province, China

**Keywords:** handgrip strength, handgrip strength asymmetry, sleeping duration, sex difference, ageing

## Abstract

**Objective:**

This study aimed to explore the potential correlation between sleeping duration and the risk of handgrip strength (HGS) asymmetry in older Chinese adults.

**Methods:**

The data of participants (65+ years of age) were obtained from the first Wave 1 (2011) of the China Health and Retirement Longitudinal Study (CHARLS). Information on sleeping duration during the previous month was collected from self-reports and was divided into three groups: long (>8 h), normal (6–8 h), and short (< 6 h). HGS was computed as the average of two tests per hand and asymmetric HGS was diagnosed when the ratio of average non-dominant to average dominant HGS was greater than 1.1 or less than 0.9. Logistic regression analyses were employed to gauge the relationship between sleeping duration and asymmetric HGS risk.

**Results:**

In total, 3,174 participants were enrolled in this analysis, of whom 51.54% (1,636/3,174) were male. The proportions of asymmetric HGS were 42.18% in males and 41.87% in females. The sleeping duration (hours) in the asymmetric and non-asymmetric HGS groups were 6 h (5,8) and 7 h (5,8) in males with a significant difference between them (*p* = 0.015), and 6 h (5,8) and 6 h (5,8) in females with no significant difference (*p* = 0.415). Compared with non-asymmetric HGS individuals, the proportions of normal, short, and long sleeping duration were 40.4, 47.3, and 37.7% in males with a significant difference (*p* = 0.023), and 42.4, 40.3, and 43.8% in females with no significant differences (*p* = 0.685). An adjusted logistic regression analysis model confirmed that short sleeping duration was significantly associated with asymmetric HGS risk among older males (*p* = 0.048, OR = 1.252, 95%CI:1.002–1.565).

**Conclusion:**

The results demonstrated that short sleeping duration (<6 h) was a risk factor for increased HGS asymmetry among older Chinese males.

## 1. Introduction

Handgrip strength (HGS) asymmetry, defined by a ratio of non-dominant to dominant HGS that is greater than 1.1 or less than 0.9, has been proposed as a marker of impaired muscle function that may help to improve HGS protocols and the prognostic utility of handgrip dynamometers (1, 2). Asymmetric HGS is common in older adults and is reported to be fairly prevalent worldwide: about 53.5% in Americans (3), 45.6% in South Koreans (4), and 45.2% in Chinese (5). Recent studies have revealed that, in older adults, HGS asymmetry was closely linked to worse health outcomes, such as chronic morbidity status (6), mortality (7), falls (8), limitations in specific activities of daily living (ADL) (2), geriatric hip fracture (9), and neurological and psychiatric disorders (5, 10). To date, the mechanism through which asymmetric HGS occurs remains unclear, although brain health as well as neural and neuromuscular functions (11, 12) have been reported to be involved. During HGS testing, neural systems responsible for controlling coordinated movements regulate the synchronous contraction of appropriate muscles, and the motor skills and synchronous muscle contractions are partly regulated by the neural system that mediates the control of coordinated movements, and the widespread asymmetric HGS reflect differences in neuromuscular system function or brain health (11).

Sleep disturbances, which encompass various factors such as duration, stages, quantity, and quality (13), have been found to affect nearly half of the older adult population (14). Research has shown that sleep disturbance in older adults is associated with negative health outcomes, including an increased risk of dementia (15), higher all-cause mortality (16), greater risk of depression (17), reduced muscle mass and weaker grip strength (18), and decline in cognitive function or aging (19, 20). A recent study discovered that higher HGS in the non-dominant hand was associated with larger hippocampus volumes (*p* = 0.02), while HGS in the dominant hand was associated with higher frontal lobe volumes (*p* = 0.02) (21). A growing body of evidence suggests that age-related changes in brain structure, such as a decrease in gray matter volume and density and reduced volumes in the hippocampus and frontal regions, are connected to sleep disturbances in older adults (22–25). Consequently, sleep disturbances may contribute to a decline in both non-dominant and dominant HGS. However, it is currently unclear whether sleep disturbances can cause a disproportionate decline in HGS between the dominant and non-dominant hands, known as HGS asymmetry.

Abnormal sleeping duration is classified as sleep disturbance and a previous study has reported that reductions in sleeping duration due to difficultly in falling asleep and trouble in staying asleep is common in older adults (26). During sleeping, proteins associated with neurodegeneration, such as β-amyloid, are cleared from the brain but a short sleep duration for one night and chronic short sleep durations could limit this function, leading to the accumulation of such proteins with potentially adverse effects on brain function (27). The accumulation of these proteins also leads to changes in both the structure and function of the brain, such as a smaller orbitofrontal cortex and precuneus (28) and cognitive decline (29). Moreover, shorter sleep durations have been reported to be linked with reduced density of slow-wave sleep activity in fronto-temporal regions with associated cortical thinning (30), possibly related to excessive wakeful neuronal activity (31). The impaired cortical thickness and abnormal function, in turn, alter the neuromuscular system function leading to asymmetric HGS (11).

Considering the interactions between short sleep duration and the brain, and that asymmetric HGS reflects a strength imbalance indicative of deficits in the functioning of the neural system or an imbalance in brain hemispheric activation (32), we speculated that there may be a correlation between asymmetrical HGS and short sleep duration during the aging process. However, to our knowledge, there is no reports on such a relationship. Therefore, this study was developed to explore the potential association of sleep duration with asymmetric HGS among older adults to better understand the risk factors of asymmetric HGS occurrence and provide some insight to reduce it.

## 2. Methods

### 2.1. Study design and participant characteristics

The data used for this study were derived from the China Health and Retirement Longitudinal Study (CHARLS), an ongoing longitudinal survey of Chinese adults over the age of 45 years. Ethical approval for data collection was obtained by the original CHARLS research team from the Biomedical Ethics Review Committee of Peking University (IRB00001052–11015). The data used in this study were from: (1) participants aged 65+ years and over from Wave 1 (2011); (2) individuals who self-reported hand dominance; (3) individuals who underwent two rounds of testing of the dominant and non-dominant hand. Participants were excluded if (1) they did not report hand dominance, (2) only had one measurement available for the dominant or non-dominant hand, (3) had missing data on sleeping duration, or (4) had incorrectly recorded data. In addition, previous studies have suggested that HGS asymmetry may reflect differences in neuromuscular system function or brain health (11). Therefore, we excluded participants with stroke and brain damage or mental retardation.

### 2.2. Sleeping time

Participants were asked the average number of hours they had slept during the past month, with all reports of sleeping time being recorded as such.

Previous studies have used classification of less than 6 h, 6 to 8 h, and more than 8 h of sleep to assign older adults into different groups (33, 34). Therefore, in this study, the subjects were also divided into three groups, namely, the normal group (6–8 h of sleep) and abnormal groups (less than 6 h representing the short-sleep group, and more than 8 h representing the long-sleep group).

### 2.3. HGS asymmetry assessment

Details of the HGS assessments used in CHARLS are described in a previous study (5). Testing was performed twice per hand. According to the updated American Society of Hand Therapist (ASHT) protocol, the average reading calculated from two tests was used to reflect HGS and HGS asymmetry was defined when the ratio of average non-dominant to average dominant HGS was greater than 1.1 or less than 0.9.

### 2.4. Covariates

Information on covariates was obtained from the Wave 1 data from the CHARLS study, including age, height, weight, marital status (married or single/divorced/widowed), education (illiterate, primary school and below, junior high school and above), dominant hand, residential area, smoking history, drinking history, physical disability, vision problems, hearing problems, hypertension, dyslipidemia, diabetes, heart problems, chronic lung disease, arthritis or rheumatism, liver disease, kidney disease, memory-related diseases, and emotional or nervous or psychiatric problems.

### 2.5. Statistical analyses

SPSS 25.0 was used for all analyses. A two-sided *p* < 0.05 was the threshold for significance. Normally distributed quantitative data are reported as means ± SD (standard deviation), whereas non-normally distributed values were reported as medians (quartiles) or median (P25, P75). Categorical variables were presented as numbers and percentages. Characteristic comparison was performed via Rank-sum and Pearson’s chi-square tests.

Logistic regression analyses were used to establish potential associations between sleeping duration and asymmetric HGS risk among enrolled participants. Due to a significant relationship observed between sleeping time and asymmetric HGS among males but not females, the logistic regression models were developed exclusively for males. Variables that showed a significant association with asymmetric HGS (*p* < 0.05) in univariate analyses were used to construct the models for male participants. Model 1 represents a non-adjusted model, Model 2 was adjusted for marital status, and Model 3 was adjusted for marital status, heart problems, chronic lung diseases, and memory-related diseases.

## 3. Results

Based on established inclusion/exclusion criteria, this cross-sectional study enrolled 3,174 participants aged and over 65 years; of these, 51.54% (1,636/3, 174) were male and 48.46% (1,538/3, 174) were female. The characteristics of the male and female participants with asymmetric or non-asymmetric HGS are provided in Table 1. The proportions of asymmetric HGS were 42.18% in males (690/1,636) and 41.87% (644/1,538) in females. Compared with individuals with non-asymmetric HGS, a significant difference was observed in terms of marital status (*p* = 0.001), heart problems (*p* = 0.005), chronic lung diseases (*p* = 0.007), and memory-related diseases (*p* = 0.022) in male participants. At the same time, BMI (*p* = 0.012), heart problems (*p* = 0.045), and memory-related diseases (*p* = 0.029) were associated with HGS asymmetry in female participants, while other covariates did not differ significantly between the two groups.

**Table 1:** Baseline characteristics of study participants stratified by sex according to asymmetric HGS.

Characteristics	Male Asymmetric HGS			Female Asymmetric HGS		
	No (*n* = 946)	Yes (*n* = 690)	*p*-value	No (*n* = 894)	Yes (*n* = 644)	*p*-value
Age, year, median (iqr)	71 (67, 75.25)	71 (67,75)	0.369	70.5 (67, 76)	71 (67,76)	0.336
BMI, kg/m^2^,median(iqr)	21.7 (19.6, 23.9)	21.4(19.7, 23.8)	0.56	22.8 (20.4, 25.6)	22 (19.8, 25.1)	0.012
Marital status, n (%)			0.001			0.652
Married	855 (59.3)	587 (40.7)		787 (58.3)	562 (41.7)	
Divorced/Widow/Single	91 (46.9)	103 (53.1)		107 (56.6)	82 (43.4)	
Education level, n (%)			0.249			0.762
Illiterate	254 (54.6)	211 (45.4)		228 (56.6)	175 (43.4)	
Primary school and below	399 (58.7)	281 (41.3)		378 (58.6)	267 (41.4)	
Junior high school and above	292 (59.6)	198 (40.4)		288 (58.8)	202 (41.2)	
Dominant hand, n (%)			0.52			0.745
Right	876 (58.1)	633 (41.9)		838 (58.2)	601 (41.8)	
Left	70 (55.1)	57 (44.9)		56 (56.6)	43 (43.4)	
Residential area, n (%)			0.87			0.682
Village	622 (58)	451 (42)		552 (58.5)	391 (41.5)	
City	324 (57.5)	239 (42.5)		342 (57.5)	253 (42.5)	
Smoking history, n (%)			0.649			0.062
No	597 (57.4)	443 (42.6)		520 (56.2)	405 (43.8)	
Yes	349 (58.6)	247 (41.4)		374 (61)	239 (39)	
Drinking history, n (%)			0.649			0.643
No	737 (58.1)	531 (41.9)		753 (57.9)	548 (42.1)	
Yes	209 (56.8)	159 (43.2)		141 (59.5)	96 (40.5)	
Physical disability, n (%)			0.153			0.069
No	922 (58.1)	664 (41.9)		867 (58.6)	613 (41.4)	
Yes	24 (48)	26 (52)		27 (46.6)	31 (53.4)	
Vision problem, n (%)			0.376			0.553
No	903 (58.1)	652 (41.9)		837 (58.3)	598 (41.7)	
Yes	43 (53.1)	38 (46.9)		57 (55.3)	46 (44.7)	
Hearing problem, n (%)			0.062			0.447
No	882 (58.5)	626 (41.5)		813 (58.4)	578 (41.6)	
Yes	64 (50)	64 (50)		80 (55.2)	65 (44.8)	
Hypertension, n (%)			0.375			0.5
No	738 (58.3)	527 (41.7)		699 (58.6)	493 (41.4)	
Yes	204 (55.7)	162 (44.3)		193 (56.6)	148 (43.4)	
Dyslipidemia, n (%)			0.335			0.292
No	847 (58.3)	606 (41.7)		814 (58.5)	578 (41.5)	
Yes	83 (54.2)	70 (45.8)		62 (53.4)	54 (46.6)	
Diabetes, n (%)			0.12			0.719
No	901 (58.3)	644 (41.7)		830 (58.1)	598 (41.9)	
Yes	48 (49.4)	39 (50.6)		54 (56.3)	42 (43.7)	
Heart problem, n (%)			0.005			0.045
No	855 (59.1)	592 (40.9)		790 (59.1)	546 (40.9)	
Yes	86 (48)	93 (52)		100 (51.5)	94 (48.5)	
Chronic lung disease, n (%)			0.007			0.77
No	864 (59)	600 (41)		798 (58.2)	572 (41.8)	
Yes	81 (48.2)	87 (51.8)		93 (57.1)	70 (42.9)	
Arthritis or Rheumatism, n (%)			0.323			0.319
No	619 (58.7)	435 (41.3)		610 (59.1)	423 (40.9)	
Yes	326 (56.2)	254 (43.8)		282 (56.4)	219 (43.6)	
Liver disease, n (%)			0.842			0.817
No	903 (58)	655 (42)		859 (58.2)	617 (41.8)	
Yes	34 (56.7)	26 (43.3)		30 (56.6)	23 (43.4)	
Kidney disease, n (%)			0.809			0.455
No	894 (57.9)	650 (42.1)		836 (58.4)	596 (41.6)	
Yes	48 (59.3)	33 (40.7)		54 (54.5)	45 (45.5)	
Memory-related diseases, n (%)			0.022			0.029
No	938 (58.3)	672 (41.7)		866 (58.4)	632 (41.6)	
Yes	7 (33.3)	14 (66.7)		5 (31.3)	11 (68.7)	
Emotional or Nervous or Psychiatric problem, n (%)			0.539			0.946
No	931 (58.1)	671 (41.9)		884 (58)	639 (42)	
Yes	13 (52)	12 (48)		7 (63.6)	4 (36.4)	

HGS, handgrip strength.

The results of univariate analyses for sleeping duration and asymmetric HGS in the participants are summarized in Table 2. The sleeping duration (hours) in the asymmetric HGS and non-asymmetric HGS groups were 6 h (5,8) and 7 h (5,8), respectively, with a significant difference between them (*p* = 0.015). The corresponding figures for female participants were 6 h (5,8) and 6 h (5,8), respectively, with no significant difference between them (*p* = 0.415). The proportions of normal, short, and long sleeping duration individuals in males were 61.72, 29.34 and 8.94% respectively, while the values were 61.15, 30.57 and 8.28%, respectively, in females. Compared with non-asymmetric HGS individuals, the proportions of normal, short, and long sleeping duration were 40.4, 47.3, and 37.7% in males with a significant difference (*p* = 0.023), and 42.4, 40.3, and 43.8% in females, with no significant difference (*p* = 0.685).

**Table 2:** Univariate analysis of sleeping time and asymmetric HGS.

Variable	Male Asymmetric HGS			Female Asymmetric HGS		
	No (*n* = 946)	Yes (*n* = 690)	*p*-value	No (*n* = 894)	Yes (*n* = 644)	*p*-value
Sleeping time, median (P25, P75)	7 (5,8)	6 (5, 8)	0.015	6 (5, 8)	6 (5, 8)	0.415
Sleeping time, *n* (%)			0.023			0.685
6-8 h	604 (59.6)	410 (40.4)		540 (57.6)	397 (42.4)	
<6 h	251 (52.7)	225 (47.3)		281 (59.7)	190 (40.3)	
>8 h	91 (62.3)	55 (37.7)		73 (56.2)	57 (43.8)	

HGS, handgrip strength.

Sleeping duration was associated with a decreased risk of asymmetric HGS, as shown in univariate logistic regression analysis (*p* = 0.011, OR = 0.934, 95%CI: 0.886–0.984, Table 3) and short sleeping duration (<6 h) was linked to an increased risk of asymmetric HGS in males (*p* = 0.013, OR = 1.321, 95%CI: 1.060–1.644, Table 3). After adjusting this model for marital status, heart problems, chronic lung diseases, and memory-related diseases, these analyses again confirmed that sleeping duration was closely related to a risk of asymmetric HGS (*p* = 0.024, OR = 0.94, 95%CI: 0.891–0.992, Table 3) and short sleeping duration (<6 h) was associated with a higher risk of asymmetric HGS in the male participants (*p* = 0.048, OR = 1.252, 95%CI: 1.002–1.565, Table 3).

**Table 3:** Correlations between sleeping time and asymmetric HGS in older males.

Variable	Model 1		Model 2		Model 3	
	*p*-value	OR (95% *CI*)	*p*-value	OR (95% *CI*)	*p*-value	OR (95% *CI*)
Sleeping time_*_	0.011	0.934 (0.886–0.984)	0.016	0.937 (0.889–0.988)	0.024	0.94 (0.891–0.992)
Sleeping time, *n* (%)						
6-8 h	–	1	–	1	–	1
<6 h	0.013	1.321 (1.06–1.644)	0.023	1.292 (1.036–1.61)	0.048	1.252 (1.002–1.565)
>8 h	0.524	0.89 (0.623–1.273)	0.413	0.861 (0.601–1.233)	0.338	0.837 (0.581–1.205)

^*^=Continuous variable; HGS, handgrip strength; Variables associated with asymmetric HGS (*p* < 0.05) in univariate analyses were used for logistic regression models construction. Model 1: non-adjusted model. Model 2: adjusting for marital status; Model 3: adjusting for Model 2 and heart problem, chronic lung diseases and memory-related diseases.

## 4. Discussion

This is the first study to investigate the association between abnormal sleep duration and asymmetric HGS among individuals 65+ years of age. The analyses revealed that, among male subjects, sleeping duration was closely linked to asymmetric HGS. Specifically, a short sleeping duration of < 6 h was associated with a higher risk of HGS asymmetry in comparison with a normal sleeping duration of 6–8 h but there was no such correlation observed for long sleeping duration. On the other hand, no relationship between sleeping duration and asymmetric HGS was detected among females. Based on these results, it is recommended that greater attention be paid to older males who sleep less than 6 h a night, as the risk of asymmetric HGS as well as subsequent adverse health outcomes could be reduced through timely intervention.

Based on the sleep time duration recommended by the National Sleep Foundation, an appropriate sleep duration for healthy older adults should be 7 to 8 h per day (35), with longer or shorter sleeping durations leading to poor health outcomes (16, 17, 19, 36, 37). During aging, sleep physiology, the circadian rhythm as well as health-related and psychosocial/behavioral factor (38) undergo normative change. Therefore, older adults usually have less than the recommended sleep duration. Indeed, in this study, a fairly high proportion of 29.84% of older adults had short sleep duration (<6 h), similar to results reported in previous studies. For example, one study that including 24,388 older Japanese adults (>60 years) reported that 27.94% of the participants had short sleep duration (<6 h) (39). While, cross-sectional and community-based study that enrolled 13,210 participants over 65 years from five counties (China, Ghana, India, Russia, South Africa) reported that 20.9% of the participants showed short sleep durations (<6 h) (40).

In the current study, the overall proportion of asymmetric HGS among all participants was 42.03%, with the proportions specific to males and females being 42.18 and 41.87%, respectively. An earlier study reported a nearly similar proportion of 45.6% in older South Koreans (4), while for older Americans, the rate was relatively higher at 53.5% (3). In these cases, population background, geographic location, nutritional conditions, amongst others, could potentially be responsible for the observed differences.

Previous studies have reported multiple mechanisms linking HGS asymmetry and physical performance. These mechanisms include muscle mass asymmetry, neurodegenerative disease (5), motoric cognitive risk syndrome (41), brain hemisphere morbidity-related dysfunction (32), and overcompensation and deficits in mechanical functioning due to acute and chronic injuries (42). Therefore, negative outcomes caused by short sleep duration such as impaired brain structure (28) and function, such as decreased cognitive ability (29), as well as decreased muscle mass and strength (18), and increased body comorbidity (43, 44) may contribute to the development of asymmetric HGS. Consistent with the authors’ assumptions, a strong correlation was observed between a short sleeping duration of < 6 h and a higher risk of HGS asymmetry among older males. However, a similar relationship was not noted in older females. While these gender-related differences cannot be satisfactorily explained, population characteristics such as chronic disease and differences in brain structure and function could potentially be relevant factors associated with these differences.

The current study has certain limitations. Firstly, previous evidences suggest that daily activities or physical exercise could improve or maintain muscle mass and strength in middle-aged and older adults with various chronic diseases (45, 46). The lack of data on physical activity leading to the analysis of physical activity and sleep duration as well as HGS asymmetry may limit the accuracy of conclusions. Secondly, the sample size was small. Thirdly, most of the covariates assessed at baseline were based on self-reported information without corresponding clinical diagnoses. Fourthly, HGS assessments were only conducted twice. Furthermore, the potential relationship between sleeping duration and asymmetric HGS was explored directly no stratification with respect to normal and low HGS asymmetry. As the study was cross-sectional, the reliability of the results requires verification and we were unable to analyze whether the reduction of dominant and non-dominant HGS is proportionate during aging. In addition, in this study, asymmetric HGS was defined as the ratio of average non-dominant to average dominant HGS. This differs from other definitions that consider the maximal non-dominant and maximal dominant HGS, and hence could have led to different results. Finally, there was no imaging data, such as functional magnetic resonance imaging, to support the results. Future studies should include prospective measurements of dominant and non-dominant HGS variations, involving a larger number of participants with accurate clinical diagnoses. Stratified analyses should be conducted, considering individuals with normal HGS, only low HGS, only asymmetric HGS, and low HGS with asymmetry. Additionally, more methods for measuring and identifying HGS asymmetry should be explored, and imaging facilities should be utilized to confirm the results.

## 5. Conclusion

The present study indicated that short sleeping duration was correlated with asymmetric HGS among older Chinese males.

## Data availability statement

Publicly available datasets were analyzed in this study. This data can be found here: https://charls.charlsdata.com/users/sign_in/zh-cn.html.

## Ethics statement

The ethical approval for the data collection was obtained by original CHARLS researchers from the Biomedical Ethics Review Committee of Peking University (IRB00001052–11015).

## Author contributions

YW: concept, literature research, data acquisition, manuscript preparation, and editing. ML: literature research, data analysis, manuscript editing and review. XC: design, statistical analysis, and manuscript review. All authors contributed to the article and approved the submitted version.
